# Comprehensive analysis of the mechanisms conferring resistance to phenamacril in the *Fusarium* species

**DOI:** 10.3389/fcimb.2025.1536532

**Published:** 2025-02-11

**Authors:** Alexander Dumbai Joe, Runze Liu, Xiao Luo, Ruqiya Syed, Farhan Aslam, Zhenying Luo, Zhitian Zheng

**Affiliations:** ^1^ School of Life Science and Food Engineering, Huaiyin Institute of Technology, Huai’an, China; ^2^ Ganzhou Vegetable and Flower Research Institute, Ganzhou, China

**Keywords:** phenamacril, *Fusarium* species, molecular mechanisms, myosins-5, resistance

## Abstract

The filamentous fungal genus *Fusarium* contains many species that cause catastrophic diseases in fruits, cereal, and vegetables. These diseases cause substantial losses in yield and contaminate affected crops with toxins. This causes huge losses in the agricultural sector and threatens human and animal health. The most efficient approach to control the *Fusarium* spp. is fungicide application. Phenamacril is a site-specific fungicide that exerts its antifungal effect on sensitive *Fusarium* spp. It is a new fungicide developed that targets *Fusarium graminearum* by inhibiting myosin-5, an important protein in fungal growth and disease development. Because of its remarkable specificity, the new fungicide phenamacril is regarded as environmentally benign. However, many research findings have reported the emergence of the resistance of *Fusarium* spp. to phenamacril in both the field and laboratory. This article comprehensively analyzes the mechanisms underlying *Fusarium* spp. resistance to phenamacril. We examine the molecular, genetic, and environmental factors contributing to this resistance. We emphasize the importance of continued research and integrating different approaches to monitoring and managing drug-resistant *Fusarium* spp. populations. Integrating current inventions to inform strategies for sustainable disease control practices, and increase plant health, and yield will contribute to ongoing global efforts to achieve food and nutritional sustainability for the world's rapidly growing population while ensuring the effectiveness of the fungicidal product.

## Introduction

*Fusarium* spp. include a broad genus of filamentous fungi that primarily thrive in the atmosphere and soil, typically associating with plants and rarely with humans. The pathogenic species of *Fusarium* are responsible for most plant infections, as they infect many sections of plants and trigger cell death. As it grows on a host plant, *Fusarium* spp. releases a variety of poisons that can harm the plant and other fungi. Following the infection of plants, extremely toxic substances alter the functions and structures of the plant and promote the growth and invasion of the *Fusarium* spp (Gurdaswani and Ghag, 2020). *Fusarium* spp. are the most destructive fungal to crop plants worldwide, particularly in Asia, sub-Saharan Africa, the USA, Europe, and Australia (Khokhar et al., 2013). The pathogens inflict significant harm on the host plant, resulting in a decline in both the quality and yield of the plant. These issues are significant since they pertain to both the assurance of an adequate food supply and the protection of food from potential hazards (Petrucci et al., 2023). Most *Fusarium* spp. primarily reside in the soil, *Fusarium* conidia can be spread through water via raindrops and irrigation systems. When dried, these conidia become airborne, making them highly suitable for dissemination in the atmosphere. Their widespread dispersion is facilitated by their ability to travel over large distances (Fernando et al., 2000; Lin et al., 2013).

*Fusarium* spp. are responsible for infecting key tropical fruit crops with one or more infections, which can be seen as a limiting factor for the sustainable cultivation of these products. In the field, disease induced by *Fusarium* spp. harms many crops and impacts the nutritional value and productivity during the pre-harvest stage of cultivation. *Fusarium* spp. induce post-harvest maladies consequently decreasing the sales potential of such economically viable fruits as the commodity will not be enticing to end-users (Zakaria, 2023). Around 30 *Fusarium* spp. affect crops of agronomic importance (**Table 1**). These fungi often found on aerial parts of plants, can either exist as one aspect of the natural microflora or perform as pathogenic microbes affecting horticulture crops, especially grain crops like sweetcorn, making them unsuitable for utilization (Lysøe et al., 2006; Schollenberger et al., 2008). *Fusarium* spp. may induce rot in seedlings, roots, and crowns, as well as in stalks and ears, at any stage of plant growth (Marasas et al., 1981; Rheeder et al., 1992; Cotton and Munkvold, 1998). Recently, several research studies have shreds of evidence that *Fusarium* spp. diseases present the most significant biological threats to numerous major crops globally. The pathogens affect vital nutritional produce like rice, wheat, maize, and soybeans, as well as key commercial crops such as bananas, coffee, and barley (Gordon and Martyn, 1997; Fones et al., 2020). *Fusarium* spp. crop infections pose major societal, economic, and trade issues worldwide and challenge a healthy diet due to their potential to minimize agricultural production and pollute plant products with mycotoxins. Moreover, losing key agriculture products due to fungal disease infection disrupts the economic stability of poorer nations relying on export revenues in the global market, consequently amplifying the scale of the food insecurity threat (Ekwomadu and Mwanza, 2023).

**Table 1:** Typical pathogenic Fusarium fungal species, their common host crops, symptoms, and related disease infections.

Fusarium spp.	Common host crops	Disease infection	Symptoms	Reference
*F. graminearum*	Banana	Crown rot	Wounds during harvesting often lead to infection, which softens and blackens before shriveling, allowing the decayed lesion to enter the fruit.	(Knight, 1982)
*F. graminearum*	Wheat and barley	Fusarium head blight (FHB)	Bleaching of the spikelet of the head begins at a single point and spreads. Infected kernels are discolored and may die prematurely.	(Brandfass and Karlovsky, 2006)
*F. proliferatum*	Asian lily plant	Root and bulb disease	Soft, rotting bulbs, pink to brown discoloration. Yellowing and wilting of the leaves.	(Prados-Ligero et al., 2008)
*F. proliferatum*	Mango	Leaf spot	Vegetative malformation or the excessive proliferation of vegetative buds is characterized by inflated axillary buds and disrupted apical dominance, leading to stunted growth and eventual plant death.	(Zhan et al., 2010)
*F. proliferatum*	Pineapple	Fruitlet core rot	Fruitlet discoloration, black spots on the flesh, dry rot, firm flesh, green fruit, sunken areas during ripening, severe infection, dry rot, and sunken sections.	(Vignassa et al., 2021)
*F. proliferatum*	hot pepper (Capsicum annuum L.)	Fruit rot	Pepper chili fruits have Soft, water-soaked lesions that may become discolored and mushy	(Rampersad and Teelucksingh, 2011)
*F. solani*	Avocado	Stem-end rot	Symptoms start with mild browning at the peduncle, followed by rolling and dark, mushy areas after a few days.	(Yaguchi and Nakamura, 1992)
*F. solani*	Papaya	Root rot	Reddish-dark pigmentation on diseased root and stem rot as well as rotting and wilting of the immature papaya plant.	(Singh and Kumar, 2015)
*F. verticilloides*	Maize	Fusarium ear rot	White or pink pigmentation. Shrunken, lightweight, and mummified kernels. Foul odor due to severe infection.	(Boutigny et al., 2012)
*F. verticilloides*	Mango	Leaf spot	Spots or patches on the leaves that appear discolored due to tissue death.	(Guo et al., 2021)
*F. verticilloides*	Banana	Crown rot	The infected tissue becomes limp and dark before withering, eventually allowing the rotting lesion to spread into the pulp	(AlvIndia et al., 2002)
*F. commune*	Chinese water plant (Eleocharis dulcis)	Fusarium wilt	Wilting of leaves and stems. Yellowing of the mature leaves. Stunted growth. Brown to black streaks in the vascular tissue	(Zhu et al., 2014)
*F. equiseti*	Papaya	Fruit rot	The symptoms initially appear as round, painful plaques that later develop into small depressions. As the lesion spreads, rot and mycelium begin forming on the affected fruit's surface.	(Gupta and Pathak, 1990)
*F. equiseti*	Avocado	Stem-end rot	On mature fruits, rotting lesions first appear browning to black blotching near the end of the stem. Due to the lesion spreading, it eventually causes the entire avocado to rot.	(Nilmini et al., 2020)
*F.* sp*orotrichioides*	Avocado	Stem-end rot	rotting lesions appear with browning to black blotching near the end of the stem.	(Darvas and Kotze, 1981)
*F. avenaceum*	Wheat	Fusarium head blight (FHB)	Reduced grain quality due to infection of the kernel containing mycotoxins. Discolored kernels	(Moradi et al., 2010a)
*F. oxysporum*	Potato	Stem-end rot	Temporary or permanent Welting of the foliage. Yellowing and curling of the leaves. Decay stems, and dry, brown, or dark rot appear on the tubers.	(Aktaruzzaman et al., 2014)
*F. oxysporum*	Banana	Fusarium wilt	Symptoms include leaf yellowing, staining of vascular structures, internal symptoms at the infection site, progressing to the rhizome and pseudo stem, and mature leaves wilting.	(Buddenhagen, 2009)
*F. oxysporium*	Pineapple	Dieback	The leaves exhibit desiccation and discoloration, starting from the top (crown) and progressing towards the bottom. Clusters of diseased plants were seen.	(Green and Nelson, 2015)
*F. oxysporium*	Avocado	Stem-end rot	The rotting lesion begins to change color from brown to black at the end of the stem and mature fruit. Due to its continued advances, the whole fruit begins to rot.	(Nilmini et al., 2020)
*F. fujikuroi*	Rice	Bakanae	The symptoms include seedling decay and lengthening, grain infertility, and loss of color. This diminishes the amount of grain produced and generates detrimental chemicals such as gibberellins and fusaric acid, which pose a hazard to human health.	(Hou et al., 2018)
*F. graminearum*	Oil palm	Fusarium wilt	Yellowish mature leaves and base leaves, wilting of the palm frond, leaves turn brownish and die. Stunted growth and death of the whole plant	(Flood, 2006)

**Table 2:** Description of specific genetic mutations in some major Fusarium species that have been identified as conferring resistance to phenamacril in the myosin proteins.

Fusarium pathogens	Host plant	Disease infection	Identified point of mutations for resistance	Resistance target	References
*F. graminearum*	Wheat and other major cereal crops	Fusarium head blight	S217L or E420K	FgMyo-1	(Zhang et al., 2015)
*F. asiaticum*	wheat, maize, barley, and other cereal crops	Fusarium head blight	A135T, V151M, P204S, I434M, A577T,R580G/H,I581F,S418R, I424R, A577G, K216R/E, S217P/L, or E420K/G/D	FaMyo-5	(Li et al., 2016; Zheng et al., 2024)
*F. oxysporum*	Tomatoes, bananas, cotton, cabbage, sweet potatoes, peas etc.	Vascular/ Fusarium wilt diseases, crown	V151A, S418T, S175L	FoMyo-5	(Zheng et al., 2018; Zhang et al., 2018)
*F. fujikuroi*	Rice, tomato, banana, and watermelon	Rice bakanae	K218T, S219P or S219 L	Myosin-5	(Wu et al., 2020; Hou et al., 2018)
*F. verticillioides/ F. moniliforme*	Corn (primary host), rice, sorghum, barley, sugarcane, etc.	Fusarium root rot, fusarium stem rot, and Fusarium seedlings blight	S73L or E276K	myosin-1 FvMyo1	(Ren et al., 2020)
*avenaceum*	Cereals such as barley and wheat, legumes, and ornamental plants	Fusarium seedling blight, fusarium root decay, fusarium stem decay	K216Q, N380K	Myosin-I	(Wollenberget al., 2016; Ni et al., 2020)
*F. solani*	Legumes such as beans, peas, soybeans, vegetables such as pepper, tomatoes, pumpkins cucurbits, fruits like Banana, pineapple, and papayas	Root rot, stem rot and wilt	T218S and K376M	Myosin-5	(Mao et al., 2023)

**Figure 1:**
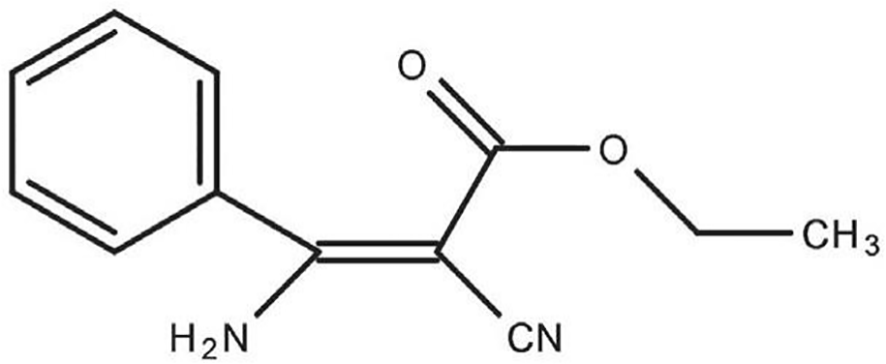
The chemical structure of phenamacril.

Chemical fungicide applications are significant for sustaining profitable crops and ensuring consistent, superior-quality outputs. Nevertheless, repeatedly using fungicides that act by similar mechanisms results in the evolution of resistance. This resistance has already appeared in many plant pathogenic species, such as the *Fusarium* spp. posing a significant threat to effective disease control (Yin et al., 2023). Phenamacril (2-cyano-3-amino-3- phenylacetic acetate, JS399–19) (**Figure 1**), a unique set of cyanoacrylate compounds that act specifically at single sites, is a fungicide specifically designed to target *Fusarium* spp. and used to control diseases caused by *Fusarium* pathogenic spp (Ni et al., 2020). This newly discovered cyanoacrylate fungicide inhibits the ATPase activity in susceptible *Fusarium* spp. myosin class I motor domains, thereby exerting its antifungal effects (Wollenberg et al., 2019). Nevertheless, the recent appearance of field and laboratory-resistant genotypes that display qualitative resistance presents a significant challenge to the further utilization of phenamacril. Nowadays, the inhibitory effects of phenamacril on *Fusarium* spp. and its resistance mechanisms have been extensively studied. As a result, research has increasingly relied on DNA sequencing, biological information systems, and genetic engineering to explore the mechanisms that regulate the actions of phenamacril and other related compounds (Zheng et al., 2024). Investigating mechanisms for resistance is essential for effective surveillance and control of resistance.

In this review article, we comprehensively explored the molecular, genetic, and environmental mechanisms conferring *Fusarium* spp. resistance to phenamacril (a crucial fungicidal agent) to ensure adequate knowledge that will enhance the efficient management of the pathogens against agronomically and economically significant crops. Exploring and integrating current research on the spread of resistance, this review endeavors to inform strategies for monitoring, managing, and mitigating resistance in crop production settings, thus contributing to more efficient and sustainable disease control practices and enhancing the efficacy of the fungicidal product.

## Phenamacril's molecular targets in *Fusarium* spp.

The molecular drivers of antifungal resistance are intricate and diverse. Recently, extensive research has been carried out on fungicide resistance focusing on the molecular basis of such resistance, and several changes in DNA sequence correlated with this resistance have been established. Although insight into these biochemical and genetic processes is essential for development despite outstanding management practices, challenges persist (Dorigan et al., 2023). A new cyanoacrylate fungicide, known as phenamacril, has been produced by the Jiangsu Branch of the National Pesticide Research and Development Center in China. It’s now available for controlling plant diseases induced by *Fusarium* spp., including *Fusarium* head blight (FHB) in *Triticum* and rice *Fusarium* wilt of rice (Yin et al., 2023). Prior studies have demonstrated that resistance of *Fusarium* spp. to the fungicide phenamacril is restricted by a single gene, FaMyo5. Diverse point mutations in this gene lead to varying levels of resistance. In addition to myosin-5, two other myosins regulate resistance to phenamacril in *Fusarium* spp (Liu et al., 2017). The protein targeted by phenamacril in the *Fusarium* spp. is myosin-5. Genetic mutations in the myosin-5 have been identified as the cause of phenamacril resistance. The evidence for these discoveries is reinforced with the observation that *Fusarium* spp. mutants, which were created using ultraviolet radiation and gene point alterations (such as K216R/E, S217P/L, and E420K/G/D) in Myosin-5, exhibit a significant level of resistance to phenamacril (Chen et al., 2008; Li et al., 2016). studies by Wu et al. (2020) revealed two-point mutations in the myosin-5 gene conferring immunity to phenamacril. One mutation occurred at codon 219, while the second occurred at codon 218, AAG→ACG.

Phenamacril has been found to prevent the germination of *Fusarium* spp. conidia and impede the growth of its mycelium. It also disrupts the activity of Myosin-5, a protein that is crucial for the production of mycotoxins in *F. graminearum* and other pathogenic *Fusarium* spp (Tang et al., 2018). Myosins are motor proteins in all eukaryotic organisms and can be classified into 35 distinct classes (Odronitz and Kollmar, 2007). This protein consists of three domains: the head or motor domain, which consists of the connection site of Adenosine Triphosphate (ATP) and actin; the tail homology domain 1 (TH1) and the SH3 domain. In *F. graminearum*, the phenmacril-resistant variants of *graminearum*, four amino acid changes were found in the motor domain (at codons 216, 217, 418, and 420), while only one mutation was detected in the TH1 domain (at codon 786). since the mutants Y2021F and YP-1 have the same mutation at codon 217, this will not lead to a substantial change in the EC_50_ value, further analysis reveals that the change in the protein sequence at codon 786 is susceptible to phenamacril (Zheng et al., 2015).

The consistency of revised agar assays and the laboratory-based ATPase assays revealed the influence phenamacril has on the fusarium is related to the molecular characteristics of its myosin. In *Fusarium solani*, the substitution of S217T is believed to be related to its resistance to phenamacril. However, it is a cautions substitution alteration because this nucleotide location has initially been associated with *Fusarium* spp. resistance to phenamacril ((Hou et al., 2018; Li et al., 2016). Phenamacril mainly targets myosin I motor protein in these fungi, essential for important cell functions such as mycelium growth and development. Phenamacril hinders the actin cytoskeleton by inhibiting myosin I, leading to damaged cellular processes and ultimately reducing fungi's growth and virulence. The selectivity of fungicides for *Fusarium* spp. especially *Fusarium* spp. due to its precise binding to this protein, making it an important asset for controlling fusarium-related diseases in cereal crops. Due to changes in the myosin I DNA sequence, resistance can be produced in these fungi, modifying the phenamacril binding site and reducing its efficacy. Although phenamacril is a powerful fungicide, the possibility of resistance requires careful monitoring in agricultural methods (Bai and Shaner, 2004; Zhang et al., 2020).

## The effect of phenamacril on *Fusarium* spp. growth, reproduction, and pathogenicity

The study by Hou et al. (2023) demonstrated that phenamacril exhibits a potent inhibitory influence on the growth of *Fusarium* spp. mycelium. It also decreases the pace at which their conidia (asexual spores) germinate. Furthermore, additional research indicates that phenamacril exhibited a teratogenic impact on conidia and blastotubules, resulting in an enhanced ratio of conidial germination from the center cells. It also demonstrates significant effectiveness in enhancing the sporulation amount of *Fusarium pseudograminearum*. Phenamacril has been shown to possess a greater control efficacy against *Fusarium* crown rot (FCR), root rot, FHB, and *Fusarium* wilt. Phenamacril is a newly developed inhibitor that can reversibly and non-competitively limit the activity of *Fusarium* spp. myosin I (Wollenberg et al., 2019). The evidence presented from the study by Zheng et al. (2018) also suggests that phenamacril effectively suppresses the mycelium growth of the *Fusarium oxysporum* Fo32931 strain and considerably decreases spore formation in a laboratory setting. However, the Fo47 and Fol4287 phenamacril-resistant strains of *F. oxysporum* did not yield the same results. These findings suggest that phenamacril may be the optimal option for managing *F*. *oxysporum* strains that are susceptible to it. Due to its capacity to greatly decrease spore generation, an initial infestation in the roots may be interrupted, resulting in the disappearance of wilting symptoms. The discovery of enhanced phenamacril targeting all FoMyo5 could address the challenge of controlling *F. oxysporum* pathogens, which causes vascular wilt in several host crops cultivated in the field.

Phenamacril hinders the development and sexual reproduction of Fusarium by inhibiting myosin I. Additionally, it diminishes the capacity of the *Fusarium* spp. to infiltrate and invade host tissues. By weakening the functionality of the spores, it also reduces its potential to spread to healthy plants (Chen et al., 2009). Research findings have indicated that phenamacril fungicide applications have tremendously minimized the intensity of *Fusarium* spp. disease such as FHB attributed to *F. graminearum* by reducing the mycotoxin concentration (Wang et al., 2018; Liu et al., 2016). Despite the novel ability of phenamacril to treat *Fusarium* spp. related diseases, the development of resistance against phenamacril due to continued application poses a serious challenge to crop management (Hou and Wang, 2012).

A report from the study by Zhang et al. (2017) showed that the phenamacril-resistant mutants exhibited significant impairments in multiple biological and physiological traits compared to the wild-type strains. These factors included hindered vegetative growth, impaired utilization of carbon sources, reduced tolerance to oxidizing and osmotic pressures, heightened susceptibility to cell wall and cell membrane integrity inhibitors, altered permeability of cell membranes, reduced glycerol accumulation, diminished disease activity, and affected cell structure and stability. These changes affect the phenotype of mutants that develop resistance to these inhibitors of phenamacril displaying numerous variances. Sequencing analysis revealed that the three parental samples were genetically similar. Additionally, the mutants TXR1, TXR2, BMR1, BMR2, SYR1, and SYR2 each exhibited a site-specific alteration in the sequence of nucleotide embedded in the myosin-5 gene. The inhibition caused by phenamacril in *F*. *graminearum* leads to problems in mycelial development and abnormalities in vesicle transport (Zhang et al., 2015). This impacts fundamental cellular processes and specific functions including controlling deoxynivalenol (DON) biosynthesis and toxisome development (Tang et al., 2018). Currently, the primary approach for managing FHB is using fungicides while the wheat is in the heading and flowering phases. This is mainly owing to the limited availability of wheat cultivars resistant to FHB (Paul et al., 2019). Phenamacril significantly decreased the FHB index and mycotoxin level by 80% (Chen and Zhou, 2009; Zhang et al., 2010; Yin et al., 2023).

## Mechanisms of resistance development in *Fusarium* spp. to phenamacril

Fungicide resistance is the inheritable decrease in how susceptible a fungus is to a particular chemical treatment. Fungicide resistance mutations have the potential to offer fitness advantages, increasing crop pathogenicity, but also have fitness costs, highlighting the necessity to additionally examine pathogen-host dynamics for better agricultural field management (Yin et al., 2023). The emergence of reduced fungicide sensitivity constitutes an evolutionary phenomenon within the pathogenic species. These developmental alterations might benefit or harm the pathogenic species' survivability, development, and replication in specific contexts, such as fungicide selection pressure (Ni et al., 2020). Some plant scientists have explored modes of action (MOAs) and fungicide insensitivity mechanisms on several scales, comprising intracellular, whole-organism, and population dynamics to manage fungicide resistance effectively. Mechanisms of action denote the specific cellular processes that a particular fungicide inhibits. The existing memorandum of agreement encompasses nucleic acid metabolism, cytoskeletal, and motor proteins, synthesis of amino acids and proteins, lipid metabolism for cell membrane structure and function, melanin production in cell walls, sterol production in cell membranes, cell wall formation, stimulating host plant defenses, and broad-spectrum activities fungicides, and other elements (https://www.frac.info/).

Phenamacril is a powerful fungicide targeting *Fusarium* spp. also known as 2-cyano-3-amino-3-phenylancryic acetate or JS399-19. It effectively prevents the growth of mycelia in most *Fusarium* spp. genera such as *Fusarium moniliforme*, *Fusarium avenaceum, F*. *graminearum*, and *F*. *oxysporum*. Still, it doesn’t have a similar effect on related pathogenic fungi (Li et al., 2008; Wollenberg et al., 2016). Studies have demonstrated that it specifically targets myosin I (MyoI) and hinders its ATPase activity, resulting in strong prohibitive effects against *Fusarium fujikuroi F*. *graminearum*, and *F*. *oxysporum* (Tang et al., 2018; Zhang et al., 2015). Investigation of the arrangement of atoms in the crystal structure of *F*. *graminearum* myo-1 demonstrated that phenamacril attaches to the allosteric nooks located within the actin-binding cleft. Upon binding, phenamacril causes the pocket in the structure of myosin with a closed actin-binding cleft to collapse (Zhou et al., 2020). However, the persistent use of the fungicide phenamacril has led to the development of resistance and rendered the fungicide ineffective in controlling *Fusarium* spp. related diseases. In an agroecosystem, a change in the DNA sequence (genetic mutation) has played a vital significance in the evolutionary pathway of plant disease toward insensitivity to fungicide (Chopra et al., 2013).

Earlier work by Zheng et al. (2015) indicated resistance to phenamacril in *Fusarium* spp. results from mutations in the Myosin 5 protein, which FGSG_01410.1 encodes. *Fusarium* asiaticum exhibits limited resistance to phenamacril when mutations occur at positions A135T, V151M, P204S, I434M, A577T, R580G/H, or I581F. Furthermore, the mutations S418R, I424R, or A577G were associated with intermediate resistance, while K216R/E, S217P/L, or E420K/G/D conferred strong resistance (Li et al., 2016). In *F*. *fujikuroi*, point mutations in K218T and S219P/L in Myosin-5 provided strong resistance to phenamacril in the field (Wu et al., 2020; Hou et al., 2018). In *Fusarium verticillioides*, high-level resistance to phenamacril has been attributed to single-point mutations in the myosin-1 FvMyo1, specifically the S73L or E276K mutations (Ren et al., 2020). In *F. oxysporum*, FoMyo5 motor domain mutations (V151A and S418T) cause inherent poor resistance to phenamacril (**Table 2**). Additionally, resistance to phenamacril in *F*. *oxysporum* f. sp. *melonis* is significantly conferred at point mutation S175L in the myosin 5 gene (Zhang et al., 2018). Conversely, prior research demonstrated that phenamacril coupled to FgMyo1 and hindered the ATPase activity and motor function of the wild-type FgMyo1, significantly influencing its localization at the tips of germlings in *F*. *graminearum (* Ni et al., 2020; Zhang et al., 2015; Zhou et al., 2020). The newly identified phenamacril-bound FgMyo1 structure provides a solid foundation for the development of innovative FgMyo1 inhibitors for the management of *Fusarium* spp (Zhou et al., 2020). Phenamacril's high affinity for FgMyo1 can be attributed to its extensive interaction with multiple residues in the 50kDa cleft. Since the cyanide group of phenamacril does not interact significantly with FgMyo1, except for the non-essential hydrogen bond with the S217 OH group, it is recommended that the cyanide group be used as a significant site of modification for the design of new fungicides (Ren et al., 2020). *F*. *fujikuroi's* resistance to phenamacril is due to the point mutations S219P or S219L, which reduced the affinity between phenamacril and the Myosin-5 protein. The amino acid at codon 219 is crucial for phenamacril binding, as a hydrogen bond cannot be formed between phenamacril and wild-type Myosin (Hou et al., 2018). The resistance-associated mutations can function as biomarkers that can be swiftly detected in the field using polymerase chain reaction (PCR). Contemporary molecular detection accelerates and identifies established fungicide tolerance linked to changes in DNA sequence, and their efficacy relies on the incorporation of an extensive listing of these alterations. While established changes in DNA sequence suggest an increased resistance threat to phenamacril, phenamacril-resistant field strains do not demonstrate equivalent resistance levels or identical types of point mutations. Extensive evaluation of resistance to this fungicide is necessary, as a field resistance study of phenamacril will elucidate this issue.

## Alteration in target protein contributes to resistance

Divergent changes in gene sequence encoding a specific location result in protein alterations at the enzyme the fungicide targets, diminishing fungicide affinity (di Rago et al., 1989). Numerous findings indicate specific changes at the target site of field strains in phenamacril and the other chemical groups of fungicides utilized in agroecosystems are linked to the development of fungicide resistance. Diverse fungicides with distinct modes of effect have been employed to manage fusarium diseases, including rice bakanae and FHB. The fungicides comprise carbendazim (benzimidazole fungicides [MBCs] that engage with tubulin), tebuconazole demethylation inhibitors (DMIs) that interact with CYP51), and prochloraz (DMI). Following the application of fungicides to manage *Fusarium* spp. infections in rice over several years, the pathogenic fungi are prone to developing resistance to MBCs by point mutations (namely, GAG→GTG at codon 198, TTC→TAC at codon 200, and GGC→GGT at codon 235) in the b2tub gene (Liu et al., 2020; Chen et al., 2014). Mutations in the CYP51 gene may cause alterations in amino acids, and amplification of CYP51 in Fusarium species confer resistance to DMIs (Sewell et al., 2017; Sun et al., 2011). A study into the cause of phenamacril resistance in *F. fujikuroi* strains, through the amplification and sequencing of the myosin-5 gene fragments, reveals that FFJX17-08R and FFJX17-09R both exhibit a point mutation from TCA to CCA at codon 219, leading to a substitution of serine with proline (S→P) in Myosin-5; this amino acid alteration has been documented previously. Furthermore, researchers analyzed the myosin-5 sequences of genes from FFSX18-02S and FFSX18-54S in comparison to that of *F. fujikuroi* (GenBank HF679023.1), identifying a point mutation (AAG→ACG) at codon 218, which leads to a lysine to threonine (K→T) alteration in myosin-5 from FFJX18-14R and FFJX18-76R (Wu et al., 2020; Hou et al., 2018).

The Fungicide Resistance Action Committee (FRAC) and the European and Mediterranean

Plant Protection Organizations (EPPOs) have documented and disseminated details regarding pathogen species resistance to various groups of fungicides (Hawkins and Fraaije, 2021; EPPO, 2023). Many fungi contain ATP-binding cassette (ABC) transporters of drugs that facilitate multidrug resistance to fungicides in laboratory mutants. Comparable mutations exhibit minimal field resistance to most fungicide groups (de Waard et al., 2006). In the enhancement of the fungicide mechanism of resistance to pathogenic fungi, ATP-binding cassette (ABC) transporters serve as a fundamental component of the target protein alternations. The movement of several substances (via active transport) such as fungicides through the membrane of the cells is ensured through ABC transporters membrane proteins (Lee et al., 2023). Efflux of the fungicides out of the cells of the fungal is the key process by which ABC transporter can confer resistance. This process minimizes the strength of the drug and obstructs it from getting to the targeted cells (Chen et al., 2023). The performance of the fungicide will weaken due to its vigorous removal, subsequently permitting the survivability and reproduction of the fungus pathogens regardless of the treatment interventions. Moreover, overexpression of the ABC transporters contributes to resistance in fungi. Efflux action takes place at an increasing level due to overexpression, which is usually initiated by alteration in the genes responsible for influencing transcription of the ABC transporter genes and also continual exposure to fungicide (Plaszkó et al., 2021). The fungus is also able to efficiently get rid of the fungicide and its toxic effects as a result of such strengthened regulation. Furthermore, any alteration within the ABC transporter genes can change the protein’s performance and particularity of the underlying substrate. Changes in those transporter genes ensure that the transporters identify and eradicate huge quantities of fungicides, enhancing resistance to multiple drug treatments, or raising the degree of compatibility of the transporters to the molecules of the fungicide. This creates an extra effective efflux mechanism (Cui et al., 2015).

Resistance to one class of fungicide confers similar resistance to another due to cross-resistance connected to ABC transporters. A single ABC transporter has to efflux numerous category fungicides to cross-resistance. This has made it difficult to manage the infections caused by fungi because of diminishing the effectiveness of accessible fungicide treatment (Ramos-Martín and D’amelio, 2023). Fungal resilience to fungicides, avoiding the buildup of toxic substances, and transportation of harmful substances that fail to exit the cell are all promoted when ABC transporters detoxify the fungicide (Sánchez-Torres, 2021). The structure of the membrane lipids, modifying the permeability of the fungal cellular membrane to chemical is controlled by alteration in ABC transporters. This alteration has the potential to diminish the infiltration into the cell of the fungal and arrive at the intracellular targets. Additionally, environmental factors such as fungicides' existence are altered by the expression and action of ABC transporter. The cells of the fungal might boost the production of ABC transporters as a means of defense, thereby strengthening their tolerant ability following fungicide encounters (Hung et al., 2024). Resistance control mechanisms of fungicides have essential bearings. To track the emergence of resistance promptly, early monitoring of the indications of the ABC transporter levels in fungi is significant (Morales Avila, 2024). Another strategy to manage resistance is by obstructing the role of efflux and recovering the efficacy of the fungicide which is achieved by improving the inhibitors that hinder ABC transporter (Wijayawardene et al., 2023). A promising approach to lowering the advancement of resistance is by interchangeably utilizing fungicides that have different modes of fungicidal infection that decrease the particular influence leading to overexpression or Mutation of ABC transporters (Buters et al., 2024).

Numerous ABC transporter genes have been identified, and their manifestation is elevated in response to different intrinsic and manufactured hazardous substances, including phytoalexins and pharmaceuticals (Amselem et al., 2011). Despite the production of efflux transporters occurring in limited time, post-drug exposure, this 'resting phase' is sufficient for many toxins to permeate the pathogen's cells, hence restricting growth. Constitutive expression of the relevant transporters inhibits drug absorption, hence eliminating the 'lag period' and imparting their insensitivity to pesticide treatment (Hahn and Leroch, 2015). A fundamental attribute of ABC drug transporters is their limited substrate particularity, allowing them to transport a diverse array of structurally distinct molecules. Seven subfamilies (A–G) of the ABC transporter are categorized according to the protein's structure (Hu and Chen, 2021).

## The role of efflux pump in lowering intracellular levels of phenamacril

Efflux pumps, mainly involving myosin-5 mutations that impact fungicide transport and sensitivity, are important in the emergence of phenamacril resistance in *Fusarium* spp. populations. Myosin-5 protein, which is essential for the cellular transport systems might release antifungal drugs like phenamacril, and undergo structural alterations as a result of these alterations. These alterations decrease phenamacril's propensity for binding to myosin-5, which hinders the fungicide's effectiveness (Hou et al., 2018). By decreasing the effectiveness of the fungicide, efflux pumps demonstrate a vital role in diminishing the intracellular level of phenamacril. The drug is vigorously transferred out of the fungal cell by these efflux pumps (membraneous proteins), thereby blocking phenamacril from getting to its intracellular targets like the myosin, which is crucial for the growth and structure of the fungal (Smith et al., 2022). Increase resistance against phenamacril fungicide due to upregulation of the efflux pump genes. These intensified activities of the efflux pump gene ensure that an eventual increase in the doses of the fungicide becomes less efficient in inhabiting the fungal growth and reproduction (Branco et al., 2023). Furthermore, the quantity of phenamacril accumulated in the intracellular pores is inadequate to destabilize vital cellular activity due to efflux pump avoidance of fungicide accumulation in the fungal cell (Nammalwar and Bunce, 2024). Resistant strains are increasingly ubiquitous because the activity of efflux pumps is undermining the therapeutic capability of phenamacril and consequentially posing obstacles to controlling fungal infections (Wang et al., 2022). To design techniques that ensure the surmount of fungicide resistance by either developing efflux pump inhibitors or altering phenamacril to escape efflux mechanisms, thorough knowledge of the interaction between efflux pumps and phenamacril is vital as far as the sustainability of the fungicide in controlling pathogenic fungal such as *Fusarium* spp. is concerned (Chen et al., 2022).

## Epigenetic change contributing to phenamacril resistance

Epigenetics modification pertains to cellular alterations influenced by causes distinct from mutations in DNA or protein sequences. In plant mycology, epigenetic processes have recently emerged as crucial regulators of antifungal drug resistance, and recent studies have thoroughly examined these mechanisms in pathogenic fungi (Patra et al., 2022; Chang et al., 2019). There is an increasing recognition of epigenetic processes influencing acquired antifungal medication resistance. A well-characterized chromatin modification entails inserting or deleting acetyl groups to or from the residues of lysine of histone tails or other protein molecules in cells (Li et al., 2015). Epigenetic alterations can be divided into two primary methods. Pathways based on RNA and chromatin. RNA-based processes involve RNA interference (RNAi) and noncoding RNAs. RNAi relates to a genetic regulatory framework by double-stranded RNA (dsRNA) induced gene reliant on homology deactivation in biological species (Moazed, 2009).

DNA methylation found specifically in the promoter areas of the genes is an essential mechanism for either suppressing or stimulating genes capable of detoxifying phenamacril and its pathways related to resistance (Yin et al., 2023). Besides histone alteration such as acetylation and methylation, modifying the chromatin system subsequently controls the availability of resistance genes. The enhancement of the drug-efflux pumps or the inhibition of the target enzymes, which decreases phenamacril effectiveness is driven by this alteration (Han et al., 2024). Post-transcriptionally, gene manifestation is controlled by non-coding RNAs like microRNAs. Targeting genes vital for phenamacril resistance mechanisms, these microRNAs break down mRNA or restrict their translation (Chen et al., 2023). Additionally, ecological vulnerability to phenamacril contributes to hereditary epigenetic modification thereby permitting the fungal community to swiftly adopt a resistance morphology regardless of depending entirely on genetic mutations.

The intricacy of phenamacril resistance which emphasizes the essence of examining epigenetic factors in advancing the sustainability of agricultural practices, stresses broadly the interaction of methylation, alteration of histone, and the non-coding RNA control. Yet, the epigenetic variables related to fungicide resistance in phytopathogen fungus remained mostly unexplored. Considering the results in the human genome of pathogenic fungi, it is feasible to put forward several epigenetic processes such as RNA structures and alterations of histone proteins and chromatin landscape that might contribute to controlling fungicide resistance or sensitivity. This is reasonable considering that epigenetic mechanisms seem to have played a significant role in the reaction of various eukaryotes to diverse stress situations (Patra et al., 2022).

### Fungicide resistance in agroecosystems: evolving trends and management issues

Fungicide resistance within the agroecosystem is regarded as a significant danger to food security (Fisher et al., 2012). Since the 1970s, resistance to primary categories of contemporary site-specific selective fungicides such as phenamacril in various species of plant pathogenic fungi has undermined plant disease management, restricted fungicide alternatives, or rendered them inefficient for agricultural use (Brent and Hollomon, 2007; Lucas et al., 2015). As fungicide resistance grows increasingly common, the efficacy of fungicides diminishes, resulting in heightened crop losses (Valarmathi, 2018). Consequently, fungicide resistance can significantly affect farmers' earnings and the nation's agricultural gross domestic product trading income (Corkley et al., 2022). An escalation in the frequency of sprays due to resistance may result in unnecessary fungicide application, leading to detrimental environmental consequences. Fungicides can contaminate soil and water, adversely impacting non-target creatures, hence altering ecosystems and producing ecological imbalances (Zubrod et al., 2019). Additionally, continued dependence on inefficient fungicides, by raising doses and spraying frequently to mitigate resistance, might promote fungicide resistance in other non-target organisms, affecting human and animal fungal pathogens (Fraaije et al., 2021). While fungicide resistance in plant pathogenic *Fusarium* spp. is a global concern to food productivity and security, shockingly not much is known about the prevalence and evolutionary mechanisms behind the formation and dissemination of fungicide resistance in several of the tropical agricultural ecosystems globally (Fisher et al., 2018).

The establishment of fungicide resistance in species of plant pathogenic fungi in an agroecosystem is a changing evolutionary phenomenon by which the prevalence of resistance alleles varies over time. It will probably alter the field effectiveness of the fungicide (Yin et al., 2023). These evolutionary modifications typically result in benefits or drawbacks for ensuring the survival of the pathogen, development, and replication amid fungicide selection pressure (Hawkins and Fraaije, 2018). Fungicide resistance seems to have emerged from a novel point mutation (*de novo* mutations) in the genes encoding the target location. The agroecosystem employing traditional disease management is deemed a hotspot for the establishment of fungicide resistance. several primary elements linked with conventional disease treatment led to the development and rapid expansion of fungicide resistance in the agricultural ecosystem. **(i).** Despite the practice of planting one crop or plant species on the same site year after year making management and harvesting easier, the extremely uniform and non-fragmented tropical farming landscapes foster the development and spread of fungicide resistance. The broad application of fungicides with a comparable mode of effect on a homogeneous and well-connected cropping region over numerous seasons creates substantial selection stress that promotes the survival and propagation of resistant strains (Valarmathi, 2018; Papaix et al., 2015). (**ii**). Traditional disease control in farming frequently relies primarily on a few numbers of susceptible fungicides. Whenever these fungicides are employed regularly and solely, species of plant pathogenic fungi with developing mutations or natural existing mutations for fungicide resistance might grow by selection resulting in adaptability. Fewer fungicide alternatives can limit the opportunity to alternate or mix distinct fungicides, which can assist prevent resistance from developing (Van den Bosch et al., 2014). (**iii**). Inappropriate application tactics, such as insufficient dosages or incorrect timing, might lead to the formation of resistance. Shock from exposure to sub-lethal dosages of fungicides can produce instability in genomics in fungi, driving the evolution of fungicide resistance or related adaptive features in the ecosystems of fungal plant diseases. Sublethal concentrations of fungicides can potentially promote selection in pathogenic populations as a whole enabling resistant variants to endure and propagate. Moreover, insufficient coverage of the plants during spraying can leave particular areas neglected, promoting resistance (Brent, 1992; Gambhir et al., 2021). (**iv**). An excessive reliance on fungicides as the major means of disease management, without incorporating other control methods can promote the evolution of resistance. Integrated Pest Management (IPM) techniques, which include multiple techniques that incorporate crop rotation, introduction of resistant cultivars, agroecological practices, and biocontrol methods in management, might assist in minimizing the reliance on chemical-based fungicides solely and lessen the danger of growing resistance (McDonald and Linde, 2002; Stukenbrock and McDonald, 2008). (**v**). The low speed of creating novel fungicides, together with the accelerated emergence of resistance in species of plant pathogenic fungi, greatly complicates the situation. The restricted availability of innovative fungicides inhibits the potential to efficiently manage resistant strains and limits the alternatives for controlling diseases (FRAC, 2023; Corkley et al., 2022).

Fisher et al., 2018 found three primary variables that can impact the pace of evolution and the outcome of fungicide resistance in ecosystems of plant diseases. The primary significant contributor is the inherited genetic variability for fungicide resistance, whether as natively existing genetic variability or fungicide-driven changes that eventually wash across the fungus population. The faster appearance of alleles providing fungicide resistance in fungi seems to be connected with the use of low levels of fungicides, which can function as a genomic pressure and increase mutagenesis. The degrees of inherited genetic variability for fungicide resistance rely on the pathogen's viable population magnitude, resulting mostly from both previous and contemporary genetic variability mechanisms, involving sexual interaction and mutation rates. This also relies on the possibility for gene transfer, which can encompass localized to a global spread of fungicide-resistant strains by the trade of diseased seeds, by which novel genes and genotypic variability are introduced (Gambhir et al., 2021; Healey et al., 2016). The subsequent important driver of fungicide resistance evolutionarily is the excessive reproductive pace of the *Fusarium* fungal pathogenic organisms in the agroecosystem which is typically immediate in agricultural surroundings as a result of the abundant genetic uniformity of host crops in broad monocultures of at-risk varieties and heavy fungicide utilize (Fisher et al., 2018; McDonald and Linde, 2002). The last main evolutionary force is the distinction survival of resistant lineages under high selection stress by fungicide sprays limiting chemical variation, throughout a lengthy run of preemptive and/or empirical, and recurrent, interventions with fungicides exhibiting a similar mode of activity. The forecasts on the outcome of evolution regarding fungicide resistance in field samples of plant diseases rely on the adaptable expense of mutations that result in resistance (Fisher et al., 2018).

### Horizontal gene transfer propagates resistance genes in *Fusarium* spp.

The emergence of genomic technologies has transformed the examination of fungal ecology and evolution. Unexpectedly, evolutionary genomic studies have demonstrated that HGT—the transfer of genes across organisms through mechanisms other than sexual reproduction—significantly impacts both the ecology and the evolution of fungal genotypes (Gonçalves et al., 2024). HGT necessitates both external environmental influences and inherent physical and molecular characteristics of the host organism. Foreign DNA must penetrate the host cell, subsequently access the nucleus, and ultimately incorporate stably into the host genome; thereafter, the incorporated DNA must proliferate and become established throughout the population. The likelihood of each event fluctuates with time and among lineages, such as between prokaryotes and eukaryotes, as well as in eukaryotes themselves (Huang, 2013; Doolittle et al., 2003). The catalysts of HGT operate within a common habitat and exhibit specificity, enhancing stress resilience, augmenting nutrition acquisition, or conferring an evolutionary benefit.

HGT is essential for the dissemination of resistance in *Fusarium* spp. enabling the interchange of genetic material among diverse organisms and enabling these fungi to swiftly adapt to environmental pressures. HGT facilitates *Fusarium* spp. in obtaining resistance genes from bacteria, fungi, or plants, resulting in enhanced resistance to antifungal agents and environmental stresses. To create an enabling environment for the fungus to efficiently overcome host defenses, the HGT approach disseminates resistance. It concurrently augments virulence by transferring genes related to decontamination and secondary metabolite synthesis, thereby facilitating the fungus to become more efficient. The key role of HGT in conferring resistance to *Fusarium* spp. is the dissemination of substitutable elements that enhance genetic diversity and accelerate adaptation. Pathogenicity islands evolved by *Fusarium* spp. are a cluster of genes that augment virulence and resistance henceforth imposing difficulties in disease management. A typical scenario of resistance emergence is azole resistance during which the HGT permits the *Fusarium* spp. to acquire resistance genes, consequently reducing the fungicide's efficacy in farming. Considering that HGT-driven multidrug resistance diminishes the efficiency of the conventional disease control approach, it contributes to the substantial obstacles in the management of *Fusarium* spp. disease of plants. A pivotal element in the dissemination of resistance and the escalating challenges in managing fungal diseases in agriculture is the HGT and its understanding is vital to the control of plant pathogenic fungi such as *Fusarium* spp (Ma et al., 2010; Mehrabi et al., 2011).

Evidence of shifts in physical traits could also be shown in *Fusarium* spp. A genome-wide comparison of three *Fusarium* spp. (*F. graminearum*, *F. verticillioides*, *F. oxysporum* f. sp. *lycopersici*) determined lineage-specific chromosomes in *F. oxysporum* f. sp. *lycopersici* which differ in prevalence across various races of *F. oxysporum* encompassing a wide range of host particularity. We are confronting a technological future characterized by changing diseases, and similar to antibiotic resistance, antifungal resistance is increasing. The process of gene transfer among fungi is crucial for the well-being, nutritional security, and increasing application of bioengineered industrial organisms (Fitzpatrick, 2012).

### Fitness costs related to fungicide resistance in *Fusarium* spp.

Fungicide resistance might confer adaptation benefits to crop infections resilient in the field, encompassing increased pathogenicity than the wild-type strains. Nevertheless, a few studies of flora diseases have demonstrated pesticide resistance and better fitness. However, it is vital to determine if fitness expenses mitigate these gains in the absence of fungicides. Resistance mutations could be considered a fitness cost in plant diseases, leading to fitness trade-offs (Hawkins and Fraaije, 2018). Theoretically, fitness can be described as the survivability and reproductive achievement of single or multiple organisms who possess a specific feature of tolerance to fungicide represented as offspring contributions to the following population (Pringle and Taylor, 2002). Considering a given isolate, the fitness elements like growth pace and severity may signify the possibility of the isolate being pathogenic. Additional variables, reproductive spore generation, and emergence, may reveal the isolate's potential to form diseases, reproduce, and propagate when growing crops. Isolates with better fitness might be related to a progressive compensating mechanism. In such a manner, it might lower the fitness costs from a nucleotide substitution in the gene of interest that confers antifungal drug insensitivity (Zur Wiesch et al., 2011).

Measuring fitness costs directly in *Fusarium* spp. populations are tedious due to the necessity of conducting *in vitro* tests with several strains, which is burdensome. Currently, DNA-based testing tools provide more economical alternatives for examining fitness costs. The study of genomes is utilized to more accurately assess the fitness costs associated with mutations that confer fungicide resistance. The mutations that give resistance may impair critical physiological or biochemical mechanisms, resulting in reduced fitness of the resistant strains. Consequently, research on the impact of single nucleotide alteration on the functionality of targeted enzymes is invaluable for resistance risk assessment. The fitness cost is affected by the environmental circumstances that prevail when the crop is cultivated.

Multiple research studies indicate that the emergence of fungicide resistance is affected by fitness penalties, which are regulated by environmental conditions such as temperature, nutritional availability, and oxidative or osmotic stress, yielding varied outcomes according to the factors examined (Hawkins and Fraaije, 2018; Thind, 2022). The persistence of pathogens' adaptation to fungicides is connected to the burden of resistant mutational populations compared to susceptible ones within a pathogenic organism. In the lack of costs of adaptation, the vulnerable fungicide in the blend keeps choosing resistant isolates that ultimately contribute to low efficacy, but with a clear survival trade-off, the selection potential of a combination of fungicides is projected to decline with the minimum consequences for controlling the disease (Mikaberidze and McDonald, 2015).

Fitness is believed to also be associated with fungicide resistance in *Fusarium* spp. a primary plant pathogenic fungal group. Many research studies have indicated that isolates of *Fusarium* spp. might display a decrease in the growth rate due to the change in the target proteins (resistance mechanisms) or the manifestation of efflux pumps that inflict metabolic pressure (Torres et al., 2022) Additionally, the reproductive progress and reduced competitive capability of such isolates might be diminished in contrast to the sensitive isolates when the fungicide stress is removed (Newbold, 2021). A few investigations further show that resistance may give rise to lower pathogenicity, as mutations giving resistance might diminish the pathogen’s capacity to spread disease to its host efficiently (Talas et al, 2024). The types of fungicides and environmental influences are the key determinants of fitness cost linked to resistance. In some situations, the resistant pathogenic isolates might not endure any major obstacle specifically when the pathogens develop restorative mechanisms to lessen the fitness trade-offs (Arastehfar et al., 2020).

### Molecular techniques for resistance detection in *Fusarium* spp.

The identification of fungicide resistance is crucial for the formulation of tactics regarding fungicide treatment and disease management. Culture-based experiments typically include subjecting strains to varying dosages of a fungicide to ascertain the EC50 value (that is, the dose at which a fungicide inhibits 50 percent of fungal growth) or the lowest restrictive dosage (Russell, 2002). While culture-based assay techniques are crucial for the preliminary identification of resistance, they are often time-consuming and laborious when an extensive quantity of strains must be evaluated. In the past years, progress has been achieved in comprehending resistance mechanisms. Target protein point mutations are the primary mechanisms that give resistance of *Fusarium* spp. to phenamacril and other fungicides. Consequently, numerous molecular detection approaches have been established. Molecular approaches, which circumvent pathogen separation and facilitate increased throughput, enable the quick detection of resistance to fungicide at substantially minimal frequencies within the pathogenic spp. Diverse molecular approaches for detecting fungicide resistance, involving loop-mediated isothermal amplification (LAMP), Polymerase chain reaction (PCR), and sequencing approaches, have been effectively established for *Fusarium* spp. Botrytis cinerea, and other plant pathogenic fungi (Ma and Michailides, 2005; Ishii and Holloman, 2015; Shao et al., 2021).

Although PCR- and DNA-sequencing-focused approaches have been effectively utilized for identifying resistance to fungicide in Asia, particularly China, the cost of equipment prevents their general deployment in the actual cropping environment. Researchers in China have concentrated on the LAMP approach. LAMP depends on Bst Polymerase and an array comprising four primers to amplify the desired nucleotide selectively under uniform temperatures (Notomi et al., 2000). Furthermore, LAMP products can be observed by simply looking through the inclusion of DNA-intercalating markers that include SYBR (Pan et al., 2013). LAMP-based approaches have various benefits such as increased susceptibility, shortened duration, and easier functioning, which makes them possibly suited for immediate detection of resistivity in the field. Given that LAMP amplifies DNA at the same temperature in just a brief duration (about three-quarters an hour), this approach could be employed in-field diagnostics (Shrestha et al., 2020). The 21st century has experienced an increase in the application of molecular approaches by scientists for the detection and control of plant diseases. The identification, taxonomy, and epidemiological analysis of fungal pathogens largely rely on contemporary molecular techniques, utilizing PCR amplification of conserved genomic areas and subsequent sequencing of the PCR results (Borman et al., 2008). Species-unique PCR primers enable the detection of target nucleotides in diseased tissues. Conventional PCR constitutes one of the fundamental methods employed for the detection of fungal infections at broad, species or strain levels, relying on the degree of specificity of the DNA primers (Jeeva et al., 2010).

Furthermore, DNA/RNA-based molecular identification and detection of *Fusarium* spp. disease infections are currently employed and demonstrate superior efficacy to immunological approaches for many reasons. As each pathogen cell encompasses a complete set of proteins (nucleic acid), they can be identified at any developmental stage by DNA-based techniques. Targeted DNA probes provide the reliable detection and identification of plant diseases at both species and race levels within diseased tissues (Kumar et al., 2015). *F. graminearum*, the pathogen responsible for FHB disease in wheat, was identified by Gupta et al., 2020 utilizing the loop-mediated isothermal amplification (LAMP) technique. Reverse transcriptase is employed to generate a cDNA duplicate of the RNA sequence within the DNA duplex, facilitating subsequent amplification. This method, termed reverse Transcriptase (RT)-PCR, is currently employed to detect pathogens like *Mycosphaerella graminicola* in wheat (Guo et al., 2005), and analyze fungal gene transcription throughout disease progression. Detection and quantification of variations in mRNA production in small quantities of cells/tissue is currently possible with real-time PCR (RT-PCR) which is also designed for undertaking multiplex identification of more than one pathogen in an identical reaction (Moradi et al., 2010b).

### Approaches for field monitoring of resistance and epidemiological studies of *Fusarium* spp.

Consistent surveillance for identifying sensitivity alterations in a pathogenic spp. subjected to fungicide treatments functions as a proactive alert system for indications of potential resistance development. Monitoring is an essential component of resistance studies, as nearly all of our understanding of the development, dispersion, and consequences of resistance in the field has been acquired using comprehensive detecting efforts. Monitoring is crucial for evaluating the efficacy of implemented anti-resistance techniques. Various techniques of sampling are employed based on the infection and the host crop, ranging from traversing cropped areas in a vehicle equipped with test plants to assess the susceptibility reaction of the entire communities of fungal pathogen, to collecting reflective single pustules and individual spore-isolated strains to evaluate the spread of resistant individuals (Thind, 2022; Brent, 1992). Typically, newly spore-forming lesions are harvested for application in susceptibility testing.

When initial instances of resistance to benzimidazoles, pyrimidines, and DMIs emerged, a variety of typical diagnostic testing methods were employed to identify fungicide resistance through target spore germ-tube length or rate of germination assessments, as well as the growth of mycelial evaluations using nutritive media modified with fungicides (e.g., culturable pathogens) as well as treated foliage isolated leaf or disc assays (for host-dependent pathogens). These experiments entail exposing the mycelia or fungal spores to a singular discriminating dosage or, more typically, to a spectrum of fungicide mixture ratio, followed by the computation of the resistance factor (RF) by contrasting the inhibiting results (EC50) of trial isolates with those of susceptible isolates. Identifying baseline sensitivities of the unaffected, vulnerable pathogen species to susceptible fungicides and adjustment of selective dosage is the utmost essential stage in the diagnosis of resistance. Yet, such biological screenings are lengthy and typically require up to three to over a week until the findings are obtained. An alternative to the proportional mycelial growth technique is a computerized quantitative test employing a microplate reader for quantifying fungal growth (Thind, 2022; Raposo et al., 1995). Mycelial proliferation assays employing fungicide-modified synthesized mediums are not consistently effective for resistance tracking (Ishii et al., 2021).

A vital aspect of disease control triggered by *Fusarium* spp. such as *Fusarium* wilt, root rot, and FHB are field monitoring of resistance and epidemiology of *Fusarium* spp. Field experiments were conducted to determine how diverse crop varieties respond to *Fusarium* spp. infection, incorporating breeding targeting and promoting resistance cultivars (Sang et al., 2024). The stringency of *Fusarium* spp. disease infection is influenced by several environmental variables including temperature, moisture content, and soil vitality, which are traced in field monitoring (Kazan et al., 2012). Furthermore, knowledge of the dispersion and distribution of the *Fusarium* pathogenic spp. in different environments is gained through epidemiological studies. Understanding disease advancement and seasonal changes enlightened farmers and researchers on the mode of dispersal of *Fusarium* spp. spores through soil, water, air, or infection spread within a crop (Munkvold, 2017). The specific focus of epidemiological studies is a significant comprehension of the pathogen and host plant interaction to ascertain the genetic mechanisms of resistance and vulnerability in diverse crop species (Dean et al., 2012). The efficiency of different control measures such as rotating the crop in the field, application of fungicide, and biological controls can be achieved through field trials to alleviate the consequences of *Fusarium* spp. damage and reduce crop loss (Khan et al., 2020). Promoting the incorporation of various disease control techniques that contribute significantly to sustainable agricultural practices is the underlying focus of resistance monitoring and epidemiological studies.

### Strategies for mitigating phenamacril resistance

#### Breeding disease-resistance crop varieties

Resistant cultivars are the most effective method for controlling *Fusarium* spp. pathogenic disease, requiring access to resistant germplasm, genetic diversity, and accurate assessment methods for effective selection. Approaches for identifying enhanced lines consist of selection by phenotype through direct symptom assessment; marker-assisted selection targeting well-defined QTL; and selection for genomic traits utilizing genome-wide models for prediction (Steiner et al., 2017). Varying environmental conditions influence the resistance of plants to *Fusarium* spp. Furthermore, when combined with additional control techniques, varietal resistance will offer an intriguing and effective strategy for controlling FHB and other phytopathogenic fungi (Bencheikh et al., 2024). Plant breeding entails a lengthy dedication and significant expenditure, calling for gene variation for traits of interest and accurate genotype-identifying methods to ensure effectiveness (Buerstmayr et al., 2020).

Resistance breeding entails evaluating if host growth phases and organs' resistances are connected. This facilitates breeding by estimating levels of resistance in the initial developmental phases. Investigations have attempted to determine the relationship between resistance in young-plant phases and *Fusarium* spp. infections in adult-plant phases (Miedaner, 1997). The capacity to alter a plant's genome presents numerous chances for the swift advancement of superior varieties with specific traits, such as resistance to Fusarium and other plant-related diseases and enhanced crop output. Induced systemic resistance (ISR) and systemic established resistance (SAR) are distinct processes, however, both signify vigorous defense mechanisms in plants (Shukuru et al., 2023). Many farmers predominantly rely on fungicides to battle fungal diseases. The adverse effects of pesticides have made the creation of fungus-resistant cultivars a primary objective of breeding initiatives.

Numerous recent publications on genome editing of susceptibility genes (S-genes) or related components in the genomes of hosts and infectious organisms have demonstrated encouraging outcomes. Nonetheless, the majority of these promising outcomes regarding disease resistance have been derived from extensively researched pathosystems. The molecular insights into host-pathogen interplay are intricate and involve several host and pathogen genes; thus, the efficacy of genome editing for disease resistance is heavily dependent on comprehending pathogen physiology and host-pathogen interactions (Raghurami et al., 2022). Exceptional disease control techniques and genetic resistance have significantly improved crop yields. Farmers desire regular gains in new cultivars to feed a growing population. Understanding the relationship between disease resistance breeding and adaptability and productivity selection is crucial for preserving and enhancing yields. Varietal resistance to pests and diseases is a paramount focus in plant breeding, aimed at reducing pesticide use, addressing emerging diseases, and strengthening the future viability and sustainability of the production of crops (Summers and Brown, 2013).

### Application of microbial populations and biocontrol agents

In agricultural settings, the use of microbial populations exhibits promise in reducing phenamacril resistance. Studies show that some microbial strains, especially when combined with organic fertilizers, can inhibit antibiotic-resistant bacteria (ARB) and lower the number of antibiotic-resistance genes (ARGs) in soil. This method promotes sustainable farming methods in addition to improving soil health (Zhao et al., 2024). Understanding the interactions between the pathogen and beneficial microorganisms as well as the mechanisms of resistance is essential to optimizing microbial populations to reduce *Fusarium* spp. resistance against phenamacril. Certain microbial communities in suppressive soils have been shown to both increase plant tolerance to *Fusarium* spp. and compete with or hinder the disease. By causing plant resistance and engaging in resource competition, species including Bacillus, Pseudomonas, and Streptomyces are useful in controlling *Fusarium* spp. infections. Crops tolerance to pathogens can be increased by using the various microbial communities found in soils that inherently inhibit *Fusarium* spp. infections (Todorovic et al., 2023).

Genetically modified specialized microbial communities modulate the emergence of antifungal drug resistance. These changes often occur as a result of changes in key genes involved in antifungal targets. This results in decreased sensitivity and increased survival advantage in the environment being treated. Although these microbial communities exhibit considerable resistance strategies, it is critical to acknowledge that not all communities display an equivalent degree of resistance. Certain environments, such as those associated with arable crop cultivation, may function as areas of diminished resistance evolution, indicating that agricultural methodologies can significantly affect resistance patterns (Fraaije et al., 2020).

### Fungicides combination treatments

The application of susceptible fungicides in combination or rotation with fungicides exhibiting diverse action mechanisms, comprising multi-site acting fungicides, is a prevalent technique to prevent or postpone the development of resilience in targeted pathogens to single-site fungicides such as phenamacril. Nonetheless, there are varying perspectives concerning their comparative efficacy. Typically, combinations of two or three fungicides, whether pre-packaged or mixed in containers, are more frequently employed. In most instances, it has been shown that employing mixtures results in a slower development of resistance compared to utilizing rotations. Mixes have delivered higher efficiency particularly when a specific degree of resistance is there. Accordingly, mixes are deemed a better alternative for avoiding crop losses due to the chance development of resistance (van den Bosch et al., 2014; Matelionienė et al., 2024). Yet, in a late inquiry by Ballu et al. (2021) with phytopathogenic fungal *Zymoseptoria tritici* and fungicide combinations of DMIs fungicides, succinate dehydrogenase inhibitor (SDHI) fungicides, and carbendazim (benzimidazole) at limited doses observed that blends might find phenotypic traits with broader or numerous resistances owing to the selection pressure of unique elements. They suggested that mixes of fungicide with site-specific activity mechanisms could never necessarily be seen as reliable tactics for resistance reduction.

The emergence of fungicide resistance in crop diseases is a serious issue hampering the efficacy of various fungicides that are site-specific classes comprising benzimidazoles, phenylamides, DMIs, quinone outside inhibitors, succinate dehydrogenase inhibitors, and phenamacril (**Table 3**). Combining different types of fungicides is useful for *Fusarium* spp. controlling resistance attributed to the fundamental processes such as attacking various biological pathways, synergistic or combined impacts, minimizing fungicide resistance formation through decreased selection pressure and prolonging resistance, delivering a wider range of control tactics for resistance handling, and combining systemic and contact fungicide may provide both curative and preventive control options.

**Table 3:** Alternative fungicides to phenamacril for controlling Fusarium pathogens for which the pathogens have developed resistance.

Fungicides	Fungicide class	Target site	Major fusarium disease targeted	Risk of resistance
Prothioconazole (triazole)	Demethylation inhibitors (DMIs)	Inhibition of sterol biosynthesis thereby affecting the cell membrane	Fusarium head blight	Medium
Tebuconazole (triazole)	Demethylation inhibitors (DMIs)	Inhibition of sterol biosynthesis	Fusarium head blight/ scab in wheat	Medium
Metconazole	Demethylation inhibitors (DMIs)	Disrupts ergosterol synthesis, thereby also inhibiting sterol biosynthesis	Fusarium head blight/ scab in wheat, barley, and other cereals	Medium
fludioxonil	Phenylpyrroles	Disrupts the fungal osmoregulatory signal transduction pathways	Fusarium root rot, fusarium seedling blight	Low/medium
Mefentrifluconazole	Demethylation inhibitors (DMIs)	Inhibition of the enzyme C14-demethylase essential for the biosynthesis of ergosterol	Fusarium head blight/ scab in wheat, barley, and other cereals. Powdery mildew, rusts, and leaf spot diseases in other crops	Medium
Azoxystrobin	Quinone outside inhibitors (QoI)	Inhibition of mitochondrial respiration and targeting, particularly cytochrome bc1 complex	Powdery mildew in cereals, grapes, etc. rust in wheat and soybeans, Botrytis cinerea notably causing gray mold	High
chlorothalonil	Chloronitriles	Disrupts the integrity of the cell membrane and metabolic functions	Powdery mildew, leaf spots, and blights	Medium-High
Carbendazim	Benzimidazoles	tubulin assembly in mitosis (cytoskeleton)	Fusarium head blight/scab, Fusarium wilt, fusarium root rot and fusarium rot	High
Thiophanate-methyl	Benzimidazoles	Disrupting cell division	Fusarium head blight/scab, Fusarium wilt, fusarium root rot, fusarium rot and crown rot	High
fenarimol	pyrimidine	Inhibition of fungal growth and development by interrupting Methionine biosynthesis (amino acids and protein synthesis	Fusarium head blight/scab, Fusarium wilt, fusarium root rot and fusarium rot	Medium-high
penthiopyrad	Succinate dehydrogenase inhibitors (SDHI)	Complex II: succinate-dehydrogenase (respiration	Fusarium head blight/scab, Fusarium wilt, fusarium root rot and Fusarium ear rot	Medium-high
Pydiflumetofen	Succinate dehydrogenase inhibitors (SDHI)	Disturbs the vitality generation of the pathogen, driving the collapse of its imperative metabolic processes	Fusarium head blight in cereal such as wheat and barley, root rot, crown rot, and damping-off in different crops	Medium-high

Both science and industry have contributed to the advancement of resistance management solutions. Many solutions emphasize minimizing the application of ineffective fungicides and employing combinations of fungicides with diverse mechanisms of action. Preserving mode-of-action diversity is crucial for resistance management (Thind, 2022). An inquiry by Ebrahimzadeh and Abrinbana, 2019, examined the impact of the application of fungicide mixtures, especially those with combined effects, which can effectively mitigate resistance development in pathogen populations. In other studies, by Samoucha and Gisi, 1987, Fungicide combinations demonstrated markedly greater efficacy in managing susceptible and resistant strains than the individual ingredients, indicating substantial synergistic effects (Samoucha and Gisi, 1987).

### Integrated disease management

IDM is the best environmentally friendly approach to preventing the selection of diseases with resistance genotypes, which may escalate gradually, disseminate to adjacent regions, and incite huge outbreaks (Stukenbrock and McDonald, 2008). Integrated management, including resistant varieties, effective soil management measures, and favorable local conditions for growth, enhances the prevention of diseases. Implementing crop rotation, employing a resistant cultivar alongside crop rotation, and utilizing a tolerant variety in conjunction with crop rotation and fungicides such as phenamacril treatment result in reductions of FHB by 50%, 80%, and 92%, respectively. For example, research conducted in North Dakota also revealed that the minimal field incidence of FHB, the lowest levels of deoxynivalenol, and the maximum productivity and test weights were attained through the implementation of various management measures rather than a singular approach (Bencheikh et al., 2024);. No singular management technique can efficiently reduce *Fusarium* spp. disease severity or mycotoxin levels; however, particular strategies may be effective, particularly when environmental factors are unfavorable for disease progression since it has been established that the consistent use of Fungicides like phenamacril with single-site mode of action has been ineffective in controlling *Fusarium* diseases due to resistance.

Breeding for Resilience, coupled with chemical and perhaps cultural control measures has the capacity for sustained control of FHB. Nevertheless, breeding for FHB resistance has been difficult due to minimal genetic gains from traditional breeding and needing complementing genomic technologies to investigate and modify the available genetic materials (McMullen et al., 2008). Hence, integrated disease control measures are the most viable technique to control FHB on cereal crops and other *Fusarium* spp. diseases on crops of agronomic significance, considering the possibility of a substantial decrease in *Fusarium* pathogen sensitivity and DON concentrations. While collaboration involving cropping techniques demonstrated a synergistic impact, integrating several approaches, in areas defined by a high risk of FHB, could be deemed the extremely feasible way of minimizing FHB and contamination with DON in wheat. An identical investigation revealed the pairing of a slightly resistant variety, application of a triazole fungicide, and tillage at heading decreased DON on wheat grains infected with FHB by approximately 97% (Dweba et al., 2017). Moreover, coordinated with various management strategies, such as cultivar resistance, will give a feasible and productive control approach for FHB management against phenamacril resistance and similar phytopathogenic fungi, like grain rusts. Consequently, an integrated management approach looks to be a feasible solution (Blandino et al., 2012).

Regarding properly managing FHB and other pathogenic Fungi, Biological Control Agents (BCAs) can be integrated with other BCAs or coupled with conventional control techniques like fungicides (Bencheikh et al., 2022). To enable the creation of integrated disease prevention tactics, it is vital to utilize appropriate bioagents and fungicides for sustainable pathogen management. Soil enrichment and particular bio-control agents such as Trichoderma and Pseudomonas are also introduced in IDM which can minimize soil-borne diseases by altering the physicochemical and microbiological environment (Venkataramanamma et al., 2023). In the integrated management approach of crop diseases, biocontrol agents and synthetic fungicides can be administered in a planned manner. Fungicides and biocontrol chemicals have been established in earlier studies to be effective at lowering pathogens or boosting plant disease resistance. Several research investigations have shown higher efficacy of integrating biological control agents and chemical fungicides for the treatment of *Fusarium* wilt diseases. Therefore, chemical fungicides and biological control agents might be efficiently utilized for the control of *Fusarium* (Meyer and Roberts, 2022; Nel and Steinberg, 2007; Lishma et al., 2024).

### Future directions for enhancing fungicide effectiveness and sustainability

Future research on *Fusarium* spp. should aim to uncover the genetic mechanisms that contribute to resistance against phenamacril. By examining a wider array of mutations in myosin genes and other molecular pathways, we can gain valuable insights into how resistance develops. Additionally, looking into alternative molecular targets beyond myosin proteins could broaden antifungal strategies and help mitigate the risk of resistance. Researching fungicide combination strategies may also reveal synergistic effects that enhance effectiveness and postpone the emergence of resistance. Incorporating biological control methods, such as beneficial microbes and natural plant compounds, presents environmentally friendly alternatives to traditional chemical fungicides.

Utilizing advanced genomic and transcriptomic analyses through next-generation sequencing (NGS) techniques will be crucial for identifying changes in gene expression associated with resistance and pathogenicity. It is also important to prioritize environmental and epidemiological studies to better understand the conditions that facilitate the spread and resistance of *Fusarium* pathogens. Innovations in fungicide formulation, including slow-release or nano-encapsulated delivery systems, could enhance the stability of phenamacril and lessen its environmental footprint. Establishing global surveillance systems to monitor resistant strains and develop effective management programs will be vital for timely interventions and informed decision-making. These research avenues are critical for sustainable disease management and for safeguarding global food security in the face of fungal diseases affecting crops.

## Conclusion

Fungicides protect crops from fungi. But overuse creates resistant strains. Research reveals complex molecular mechanisms to Identify the genetic mutation conferring fungicide resistance in pathogenic fungi. Understanding these mechanisms is essential for effective management strategies. But it brings challenges. This review article emphasizes the importance of understanding the mechanisms of phenamacril resistance in *Fusarium* spp. to improve plant pathogenic disease management. It is emphasized that although phenamacril is an effective fungicide that targets a specific myosin protein important for *Fusarium* spp. growth and pathogenesis, and the emergence of resistant genotypes is a major challenge. On this basis calls for continued research on this topic. This includes integrating the findings into strategies for monitoring and managing resistance in agricultural practices. There is also increased attention to the use of non-chemical approaches, especially biological control agents, Culture control mechanisms, and biotechnology in disease management strategies to lessen the pressure of selection caused by fungicides. The effectiveness of counter-resistance methods relied heavily on farmers' readiness to put established practices into practice. The ultimate goal is to ensure sustainable and effective control of *Fusarium* spp. related diseases in crops with significant economic value contributing to food and agricultural stability and productivity.
